# Hygienic and Literary Magazine

**Published:** 1859-11

**Authors:** 


**Hygienic and Literary Magazine.**

We desire to direct attention of our readers to the Hygeinic and Literary Magazine—the continuation of the Literary Weekly, in another form.

The new form is a "Monthly," with the substitution of "Hygeinic" for "Medical," as indicating the leading character of the publication, which portion of the Magazine will be under the exclusive control of our talented and scientific friend, V. H. Taliaferro, M. D., who is already sufficiently known to fame to make it unnecessary for us to add a word of commendation.

The "Magazine" will also embrace "Literary" and "Educational" Departments, to be managed by Messrs. Deak and Malsby, who have become associated with Dr. Taliaferro in the proprietorship of the paper. These departments will, we learn, contain the productions of the best writers in the South, and the opinions and views of leading and prominent persons engaged in the great cause of education throughout the world.

It is, however, the former and distinguished character of this Magazine, in which we feel the deepest interest, the subject being one of the most vital connected with the welfare of this and future generations.

We believe that the great want of the present time is the institution of means for the arrest of the rapid physical degeneracy which has become so palpably characteristic of our day, as to be perfectly manifest to the most superficial observer. The causes upon which depend this melancholy wasting away of bodily vigor in the human family, are "legion," prominent among which is the *insane* use of alcoholic and drugged liquors, opium and tobacco, rapidly making the American people, without exaggeration, "a nation of drunkards," and before many generations, if continued, will reduce them to a nation of pigmies and idiots. In addition to this may be mentioned the almost universal use of Quack and Patent Medicines (many of which are largely made of alcohol and opium,) and last but not least, the present *school system* of the United States which seems to be a special instrument for cursing the rising generation with early death, or, failing in this, with premature old age and wretched decrepitude, at a period when the individual should be in the highest degree capable of physical and mental effort, and the enjoyment of life.

All these and many other causes of the physical and mental enervation which is becoming so characteristic of our people, will doubtless come under review, and, we would fain hope, reach large masses throughout the country and aid in arousing them from their blindness and folly.

The first number of this Magazine will be issued by the first of December, at which time the volume commences. It is furnished to subscribers at $2 per annum (invariably in advance,) and all letters and communications should be addressed to Editors of "Hygienic and Literary Magazine," Atlanta, Ga. The subscribers to the "Weekly," will be furnished with the "Monthly."

